# Supplementary figures and images for: Incomplete lytic cycle of a widespread Bacteroides bacteriophage leads to the formation of defective viral particles
Source: PLoS Biol. 2025 Mar 31;23(3):e3002787. doi: 10.1371/journal.pbio.3002787 (PMC12135933; doi:10.1371/journal.pbio.3002787)

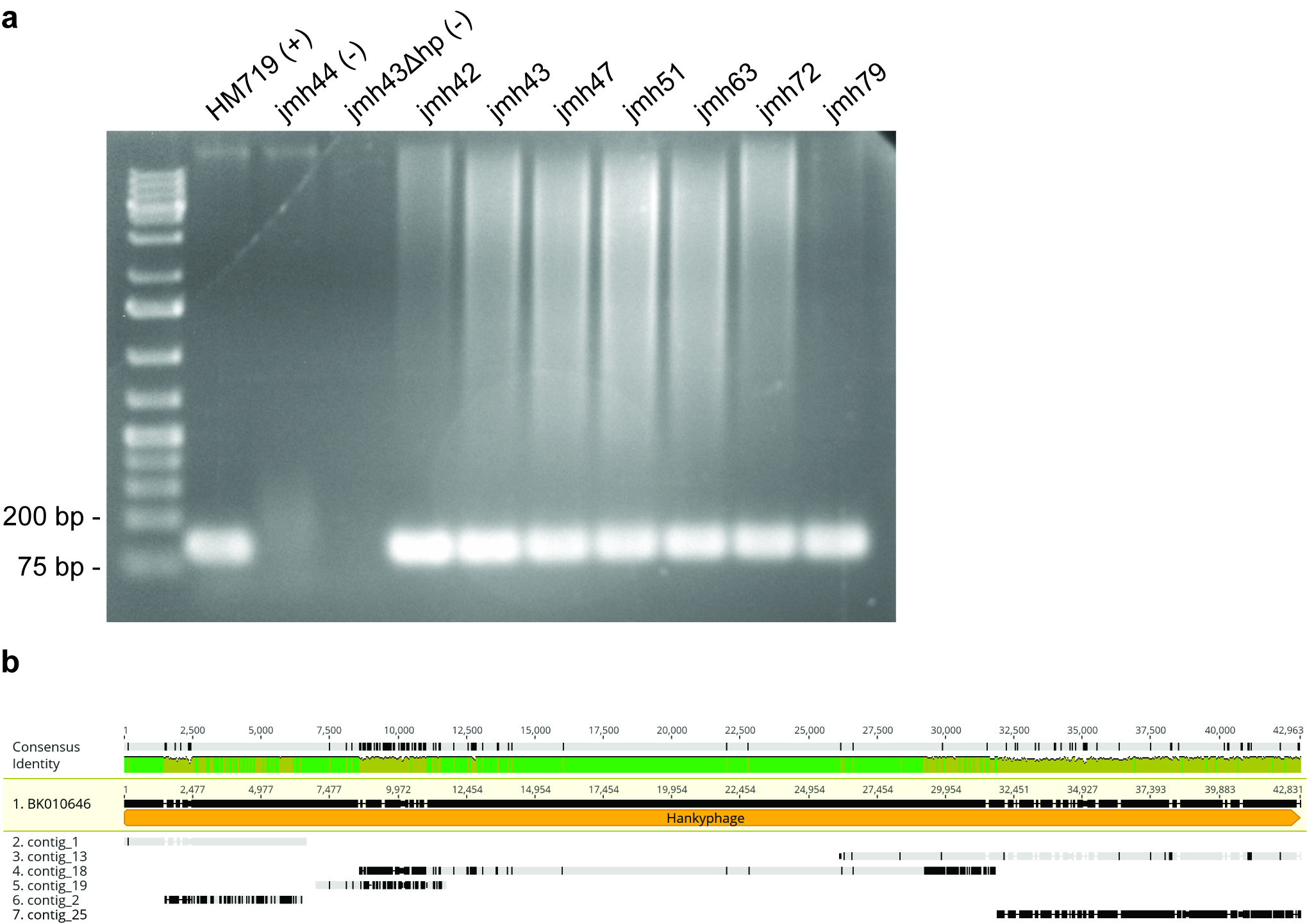

Supplement: S1 Fig — (a) Confirmation of hankyphage presence by PCR using primers located on the hankyphage reverse transcriptase (S2 Table, primers 53,54). Plus and minus signs indicate positive (+) and negative (−) controls for hankyphage DNA. (b) Alignment of the jmh63 genome on the hankyphage p00 genome using the Geneious software, suggesting that the jmh63 hankyphage coud be fragmented. (TIF) [file pbio.3002787.s001.tif]

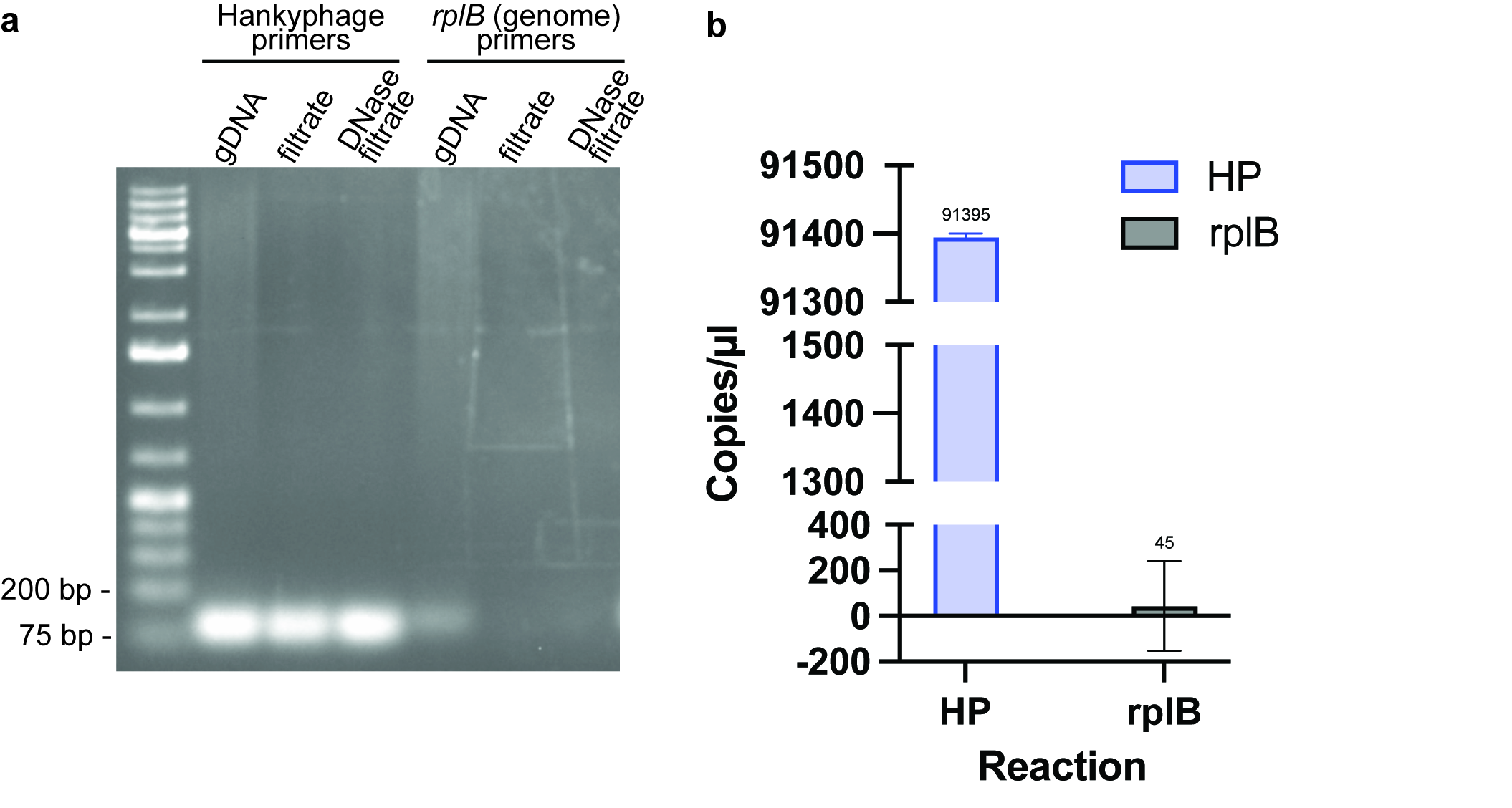

Supplement: S2 Fig — (a) Effect of DNase treatment of phage lysates using the two ddPCR primer pairs. The rplB gene is not detected after DNase treatment of jmh43 lysates. (b) Representative ddPCR result from a jmh43 DNase treated lysate. Error bars represent the uncertainty values calculated by the Naica reader. The reaction is optimized to quantify hankyphage DNA (HP) copies and has low uncertainty values. The quantification of rplB falls below the minimum concentration threshold to detect two separate clouds and has high uncertainty values, indicating low or no genomic DNA presence. The individual quantitative values underlying this figure can be found in the S5 Data file also available at: https://figshare.com/s/3ff18cc2f6cc1edab0ae. (TIF) [file pbio.3002787.s002.tif]

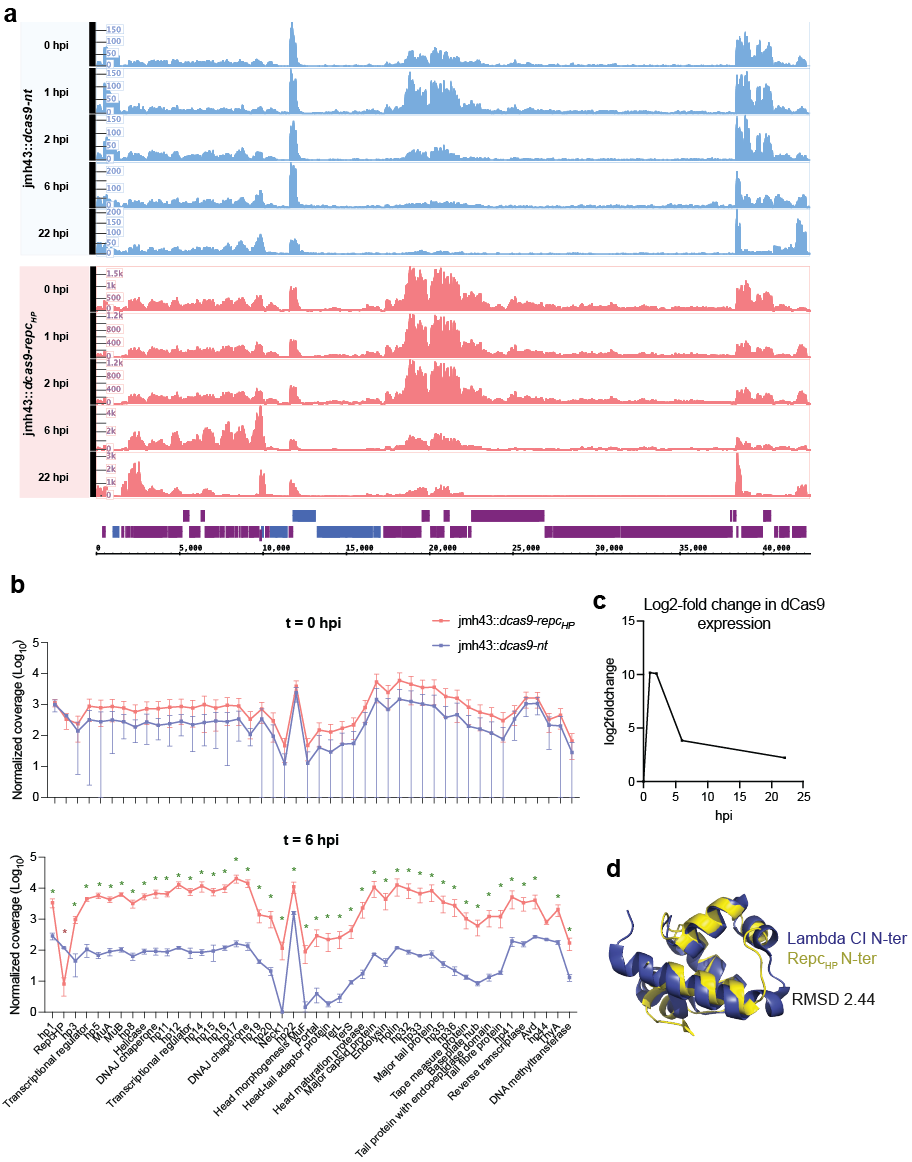

Supplement: S3 Fig — (a) Representative coverage plots of one of the three transcriptomic biological replicates at different time points of both jmh43::dcas9-repcHP and jmh43::dcas9-nt. The x-axis corresponds to the hankyphage genome, genes encoded in the positive orientation are colored in purple and those in the antisense orientation are coloured in blue. Coverage plots were produced using the Integrated Genome Browser (IGB) software. (b) Normalized transcriptomic coverage of the hankyphage genome of jmh43::dcas9-repcHP (red) and jmh43::dcas9-nt (blue) strains at different timepoints after IPTG-induced RepCHP silencing. Stars indicate significant differential up-regulation (green) or downregulation (red) of the gene in jmh43::dcas9-repcHP compared to jmh43::dcas9-nt. Coverage is normalized by total reads per sample and gene length. Values of 0 were converted to 1 for their visualization in the log10 scale. Differential gene expression analysis was performed using Deseq2. (c) Log2 fold change in dCas9 expression between jmh43::dcas9-repcHP and jmh43::dcas9-nt control. This indicates that the jmh43::dcas9-nt control expresses dCas9 less than jmh43::dcas9-repcHP. (d) Superposition of an Alphafold2 model of RepCHP (yellow) (modeled in dimer, only one monomer shown) and the crystal structure of the λ repressor CI (blue) (dimer, only a monomer shown). RMSD: root mean square of atomic positions. The individual quantitative values underlying this figure can be found in the S6 Data file also available at: https://figshare.com/s/3ff18cc2f6cc1edab0ae. (PNG) [file pbio.3002787.s003.png]

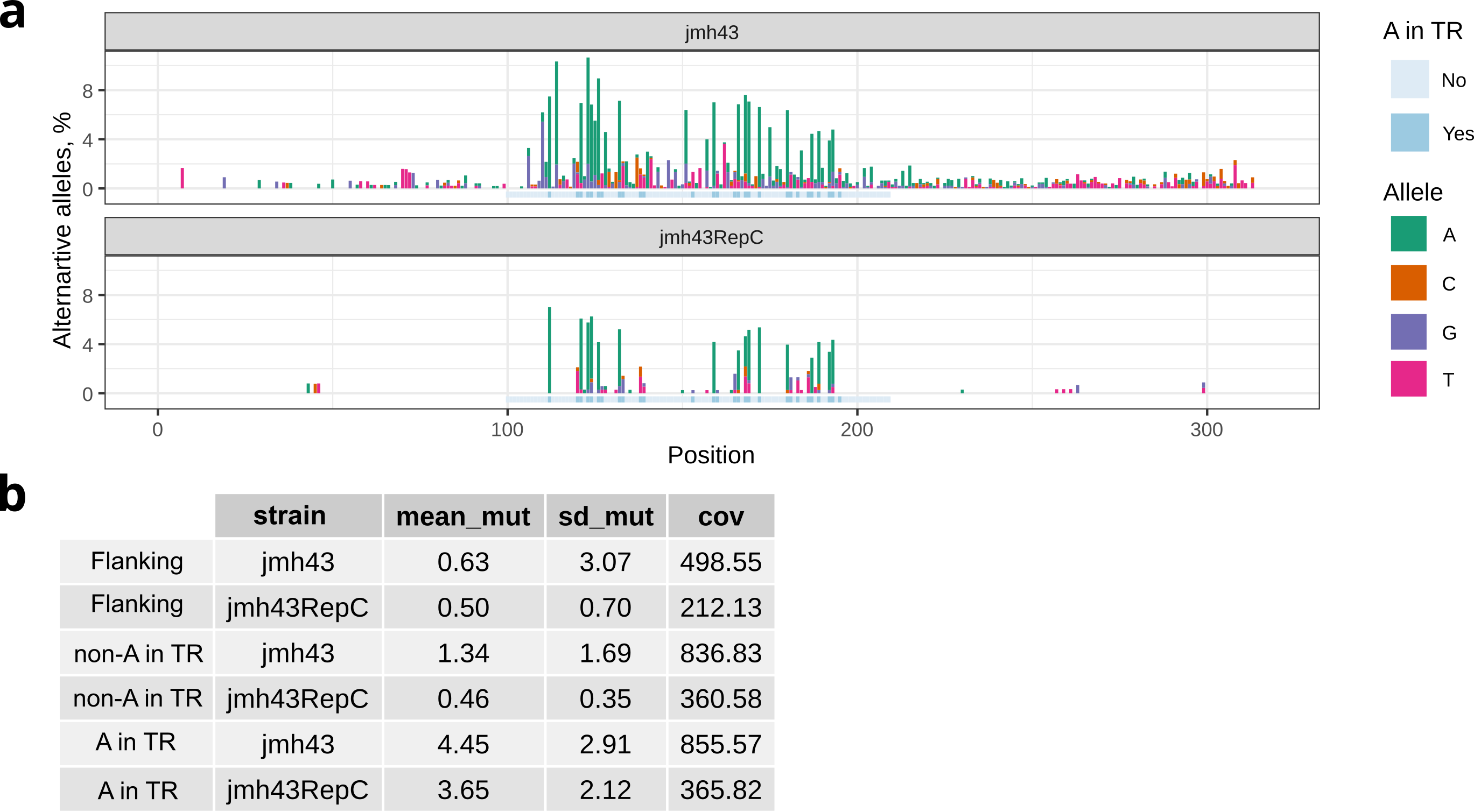

Supplement: S4 Fig — (a) Bar plots indicate the proportion of A, C, T, and G nucleotides at the VR (highlighted in blue) and flanking region which differ from the reference VR after using a global mapping approach (see methods). Of note, jmh43::dcas9-repcHP lysate was sequenced using a PCR-free kit, whereas for jmh43 a previous step of PCR amplification was required to obtain enough DNA for Illumina sequencing. As such, background mutation/noise, considerably differ between both samples. Positions targeted by the DGR system are highlighted in dark blue. (b) Summary statistics of non-reference allele data (mut) presented in panel a depending on the region considered. A in TR denotes positions expected to be diversified by the DGR compared to non-A positions. As a control, diversity around the VR flanking region (±100 nt) is also shown. Mean_mut and sd_mut denotes the average and standard deviation of non-reference allele, respectively, and coverage indicates the average mapping depth used for estimating alternative allele frequencies. Significant DGR activity was only found for jmh43::dcas9-repcHP (mean diversity in targeted positions> mean diversity in non-targeted positions + 2 sd). The individual quantitative values underlying this figure can be found in the S7 Data file also available at: https://figshare.com/s/3ff18cc2f6cc1edab0ae. (TIFF) [file pbio.3002787.s004.tiff]

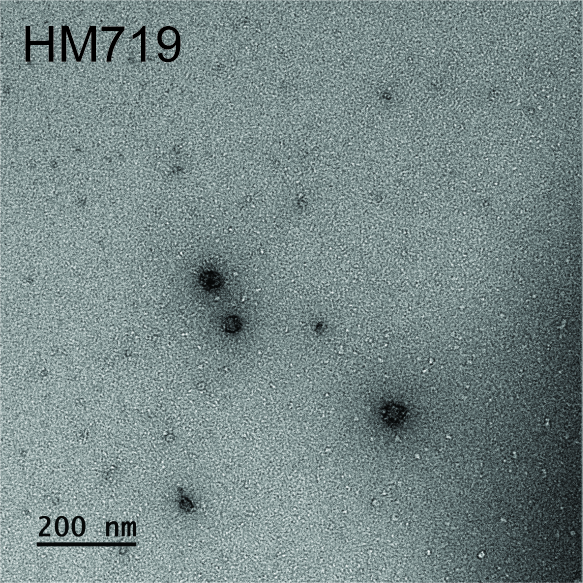

Supplement: S5 Fig — Negative staining and TEM microscopy of the reference P. dorei HM719 lysate revealed very few particles and absence of tails. (TIF) [file pbio.3002787.s005.tif]

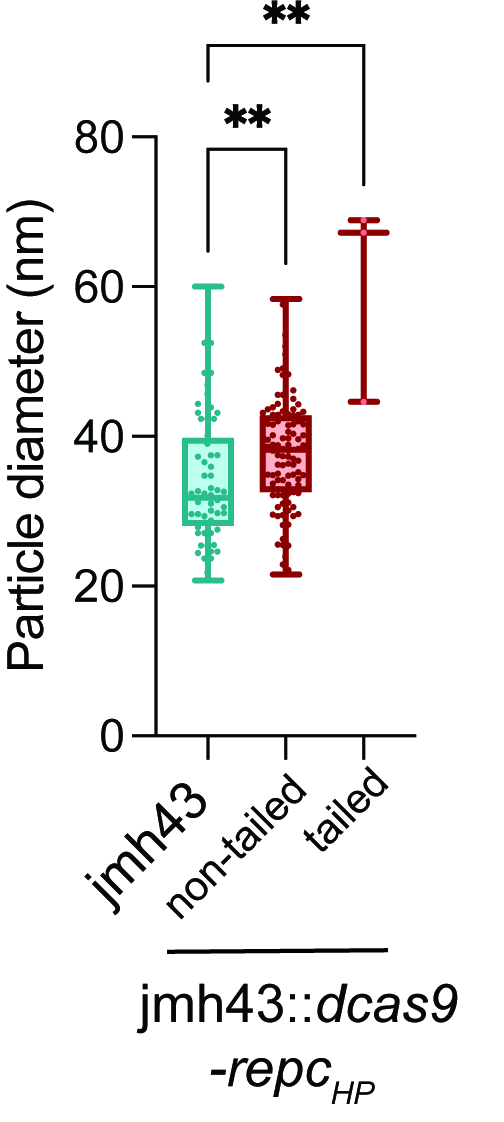

Supplement: S6 Fig — Box and violin plot showing the particle diameter from the TEM micrographs of the jmh43 and jmh43::dcas9-repcHP lysates, including tailed and non-tailed particles. Individual values shown as points, boxes indicate the median and bars indicate the minimum and maximum values. Significant differences in sizes were calculated via a non-parametric Kruskal–Wallis test and are shown as asterisks (the two asterisks correspond to a p-value between 0.05 and 0.005). The individual quantitative values underlying this figure can be found in the S8 Data file also available at: https://figshare.com/s/3ff18cc2f6cc1edab0ae. (TIF) [file pbio.3002787.s006.tif]

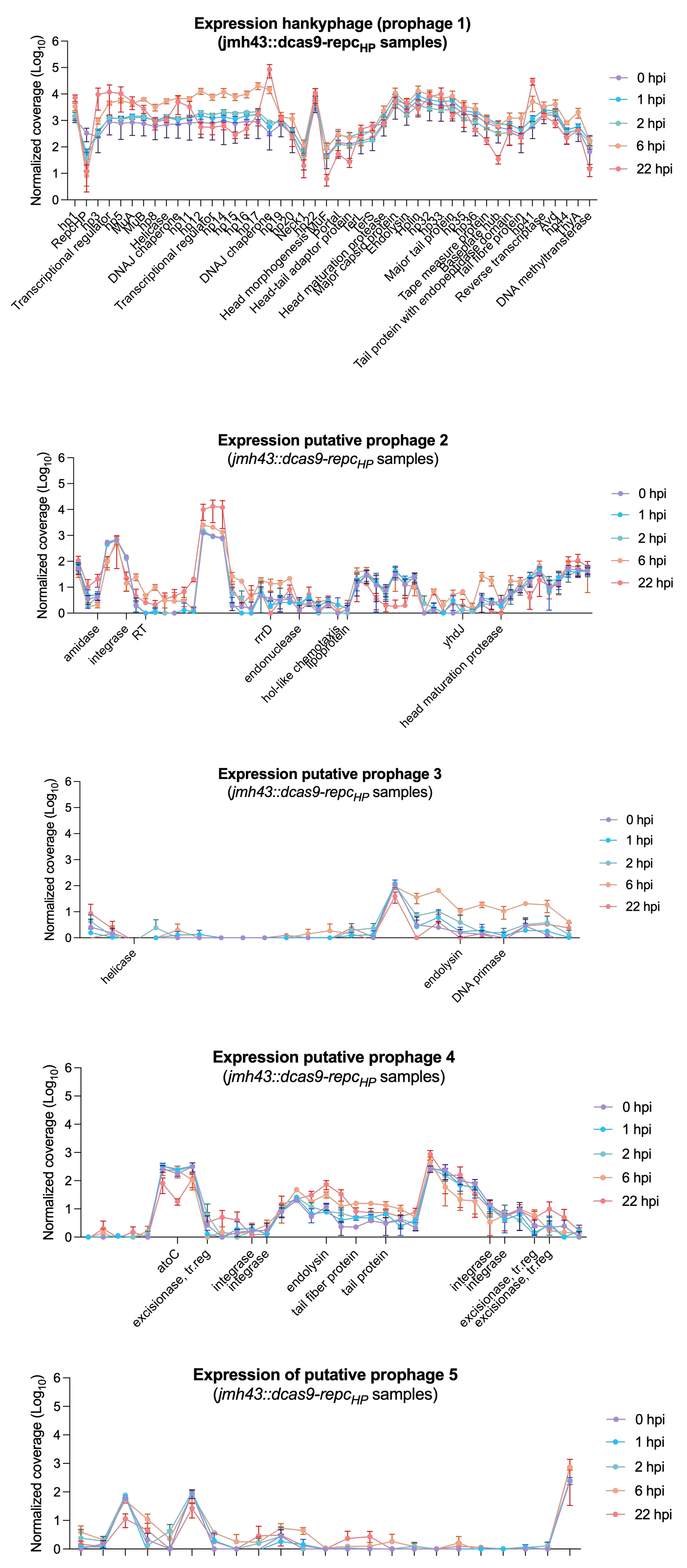

Supplement: S7 Fig — Normalized transcriptomic coverage of the hankyphage genome and other putative prophages of jmh43::dcas9-repcHP at different timepoints after IPTG-induced repcHP silencing. Coverage is normalized by total reads per sample and gene length. Values of 0 were converted to 1 for their visualization with a Log10 scale. The individual quantitative values underlying this figure can be found in the S9 Data file also available at: https://figshare.com/s/3ff18cc2f6cc1edab0ae. (TIFF) [file pbio.3002787.s007.tiff]

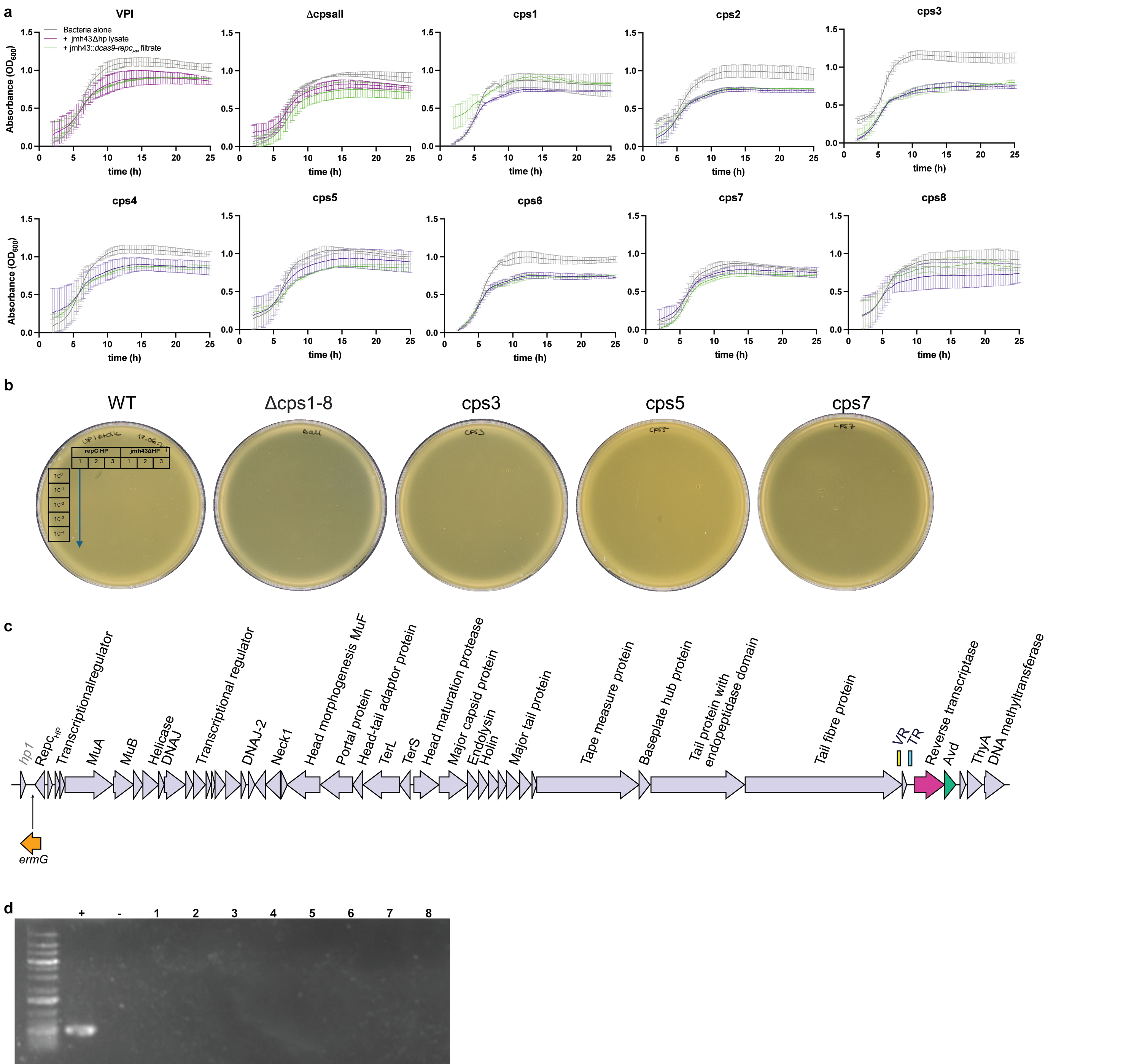

Supplement: S8 Fig — (a) Growth curves of Bacteroides thetaiotaomicron VPI5482 capsule mutants exposed to lysates of jmh43ΔHP or jmh43::dcas9-repcHP. Curves are the average of two biological replicates. The individual quantitative values underlying this figure can be found in the S10 Data file. (b) Representative spot assay photographs, overlays of different capsule mutant strains with spots of jmh43::dcas9-repcHP (RepcHP) or jmh43ΔHP lysates, no lysis plaques could be observed. (c) Illustration representing the location of insertion of ermG (orange) within the hankyphage genome within the jmh43 strain. (d) PCR checks indicating the absence of ermG acquisition of different colonies (1–8) as well as a positive (+) and negative (−) control. (PNG) [file pbio.3002787.s008.png]

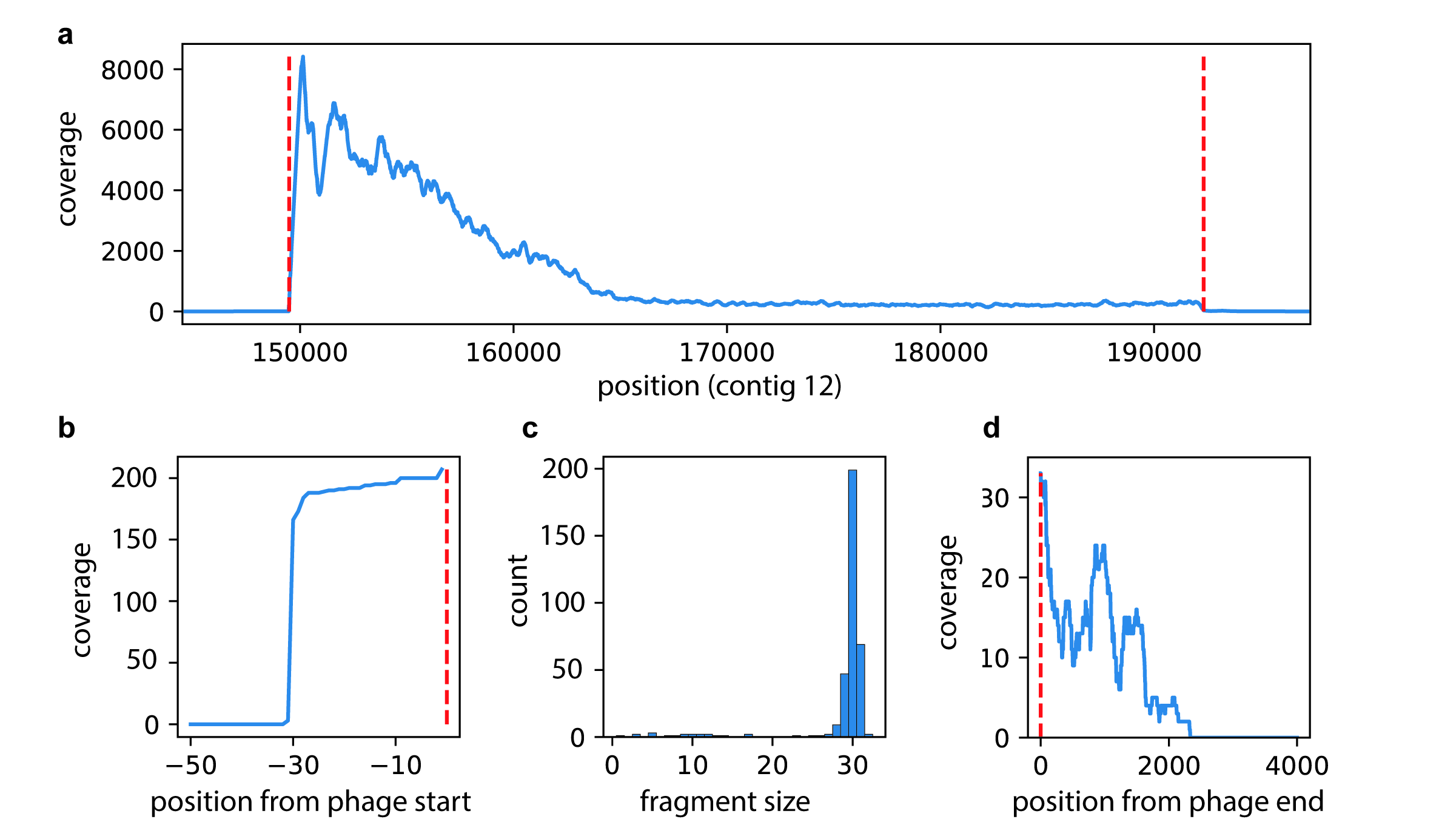

Supplement: S9 Fig — (a) Sequence coverage of DNA extracted from the hankyphage capsids after mapping to the jmh43 chromosome region where the phage is initially located. Red dashed lines mark the beginning (5′) and end (3′) of the phage. (b) Zoom in on the 5′ end of the phage. (c) Distribution of the size of chromosomal fragments packaged at the 5′ end of the phage. (d) Zoom in on the 3′ end of the phage shows decreasing coverage of jmh43 chromosome over a ~ 2,300 nucleotide region indication packaging of a variable size fragment of the chromosome on the 3′ end of the phage of approximately 1–2.3kb. The individual quantitative values underlying this figure can be found in the S11 Data file also available at: https://figshare.com/s/3ff18cc2f6cc1edab0ae. (TIF) [file pbio.3002787.s009.tif]

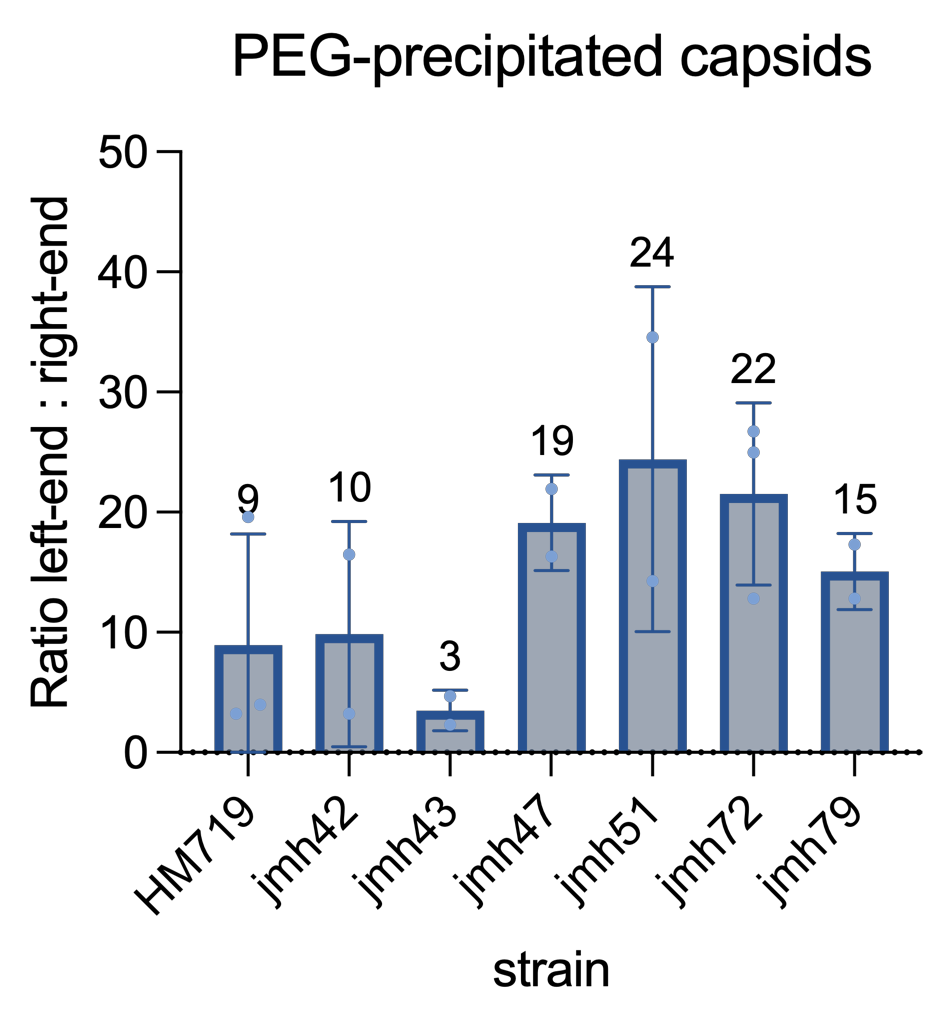

Supplement: S10 Fig — Bar plot showing the ratio of hankyphage genome right- and left-end copies quantified by ddPCR on PEG-precipitated capsids of HP+ strains. Error bars depict the standard deviation from the mean of two or three biological replicates (individual values plotted as points). The individual quantitative values underlying this figure can be found in the S12 Data file also available at: https://figshare.com/s/3ff18cc2f6cc1edab0ae. (TIFF) [file pbio.3002787.s010.tiff]

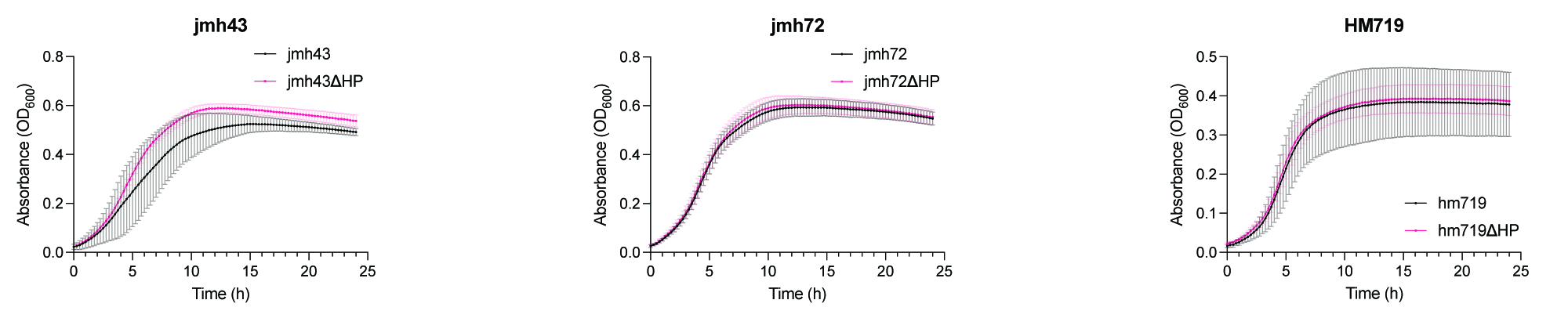

Supplement: S11 Fig — Growth curves performed on three HP+ strains and their respective mutant deleted for the hankyphage (three biological replicates). The individual quantitative values underlying this figure can be found in the S13 Data file also available at: https://figshare.com/s/3ff18cc2f6cc1edab0ae. (TIF) [file pbio.3002787.s011.tif]

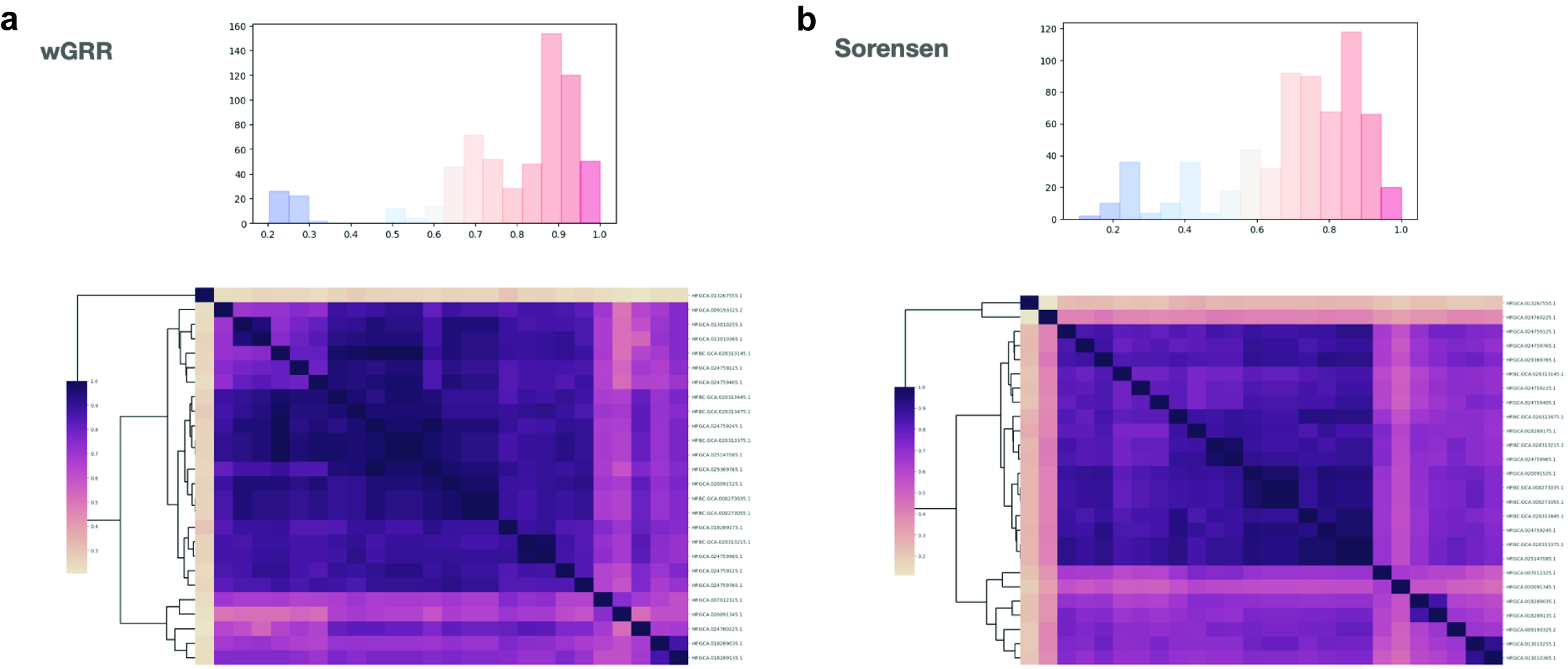

Supplement: S12 Fig — (a) wGG and (b) Sorensen-Dice matrices supporting the hankyphage phylogenetic tree from Fig Fig 4. (TIF) [file pbio.3002787.s012.tif]

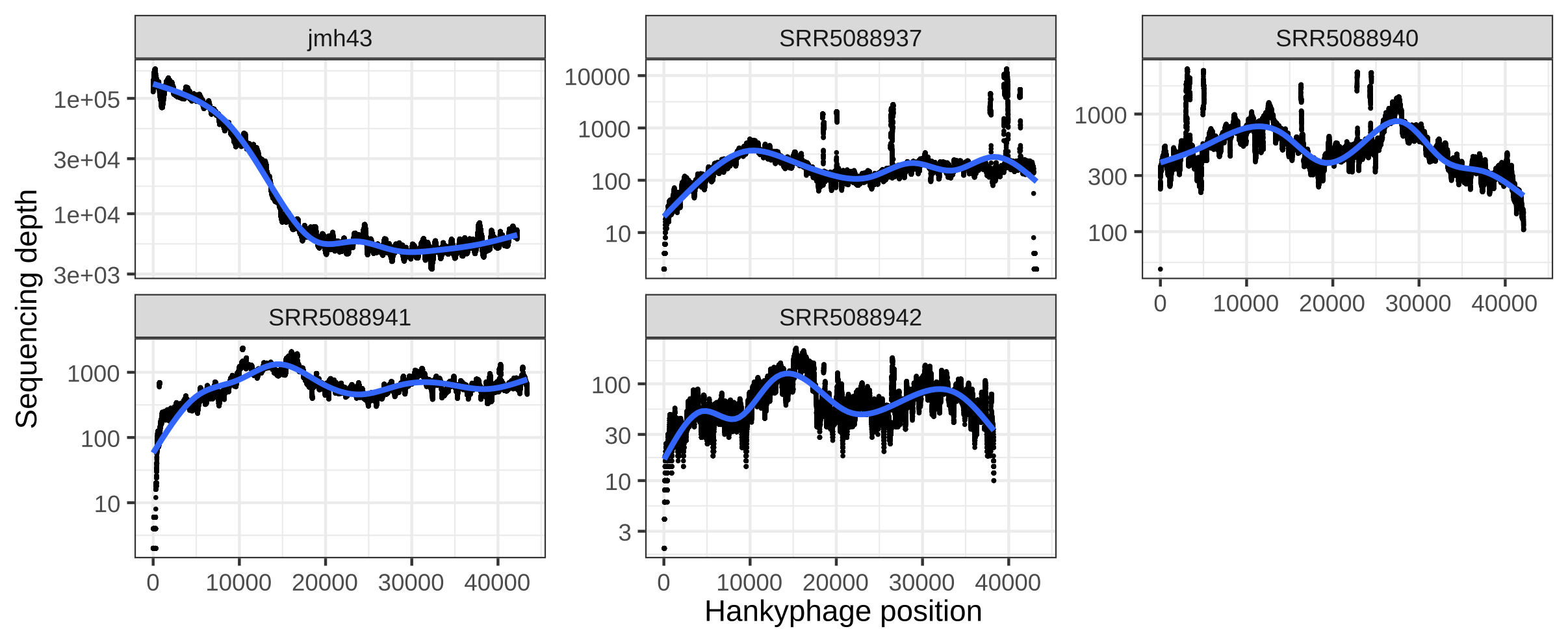

Supplement: S13 Fig — Coverage plots showing the patterns of hankyphage DNA coverage in four representative viral metagenomic samples as well as in jmh43 laboratory-extracted lysates. The gradual increase of coverage from the 5′ to the 3′ in the metaviromes may be attributed to the use of sequencing kits that use transposases (Nextera) to ligate the adapters in this study [89]. In the case of hankyphages, this might prevent the ligation of the adaptors at the phage 5′ but not at the 3′ end given the differential amount of host DNA that is packaged at each side. Coverage is normalized by the sum of the mapped reads to that region. The individual quantitative values underlying this figure can be found in the S14 Data file also available at: https://figshare.com/s/3ff18cc2f6cc1edab0ae. (TIFF) [file pbio.3002787.s013.tiff]

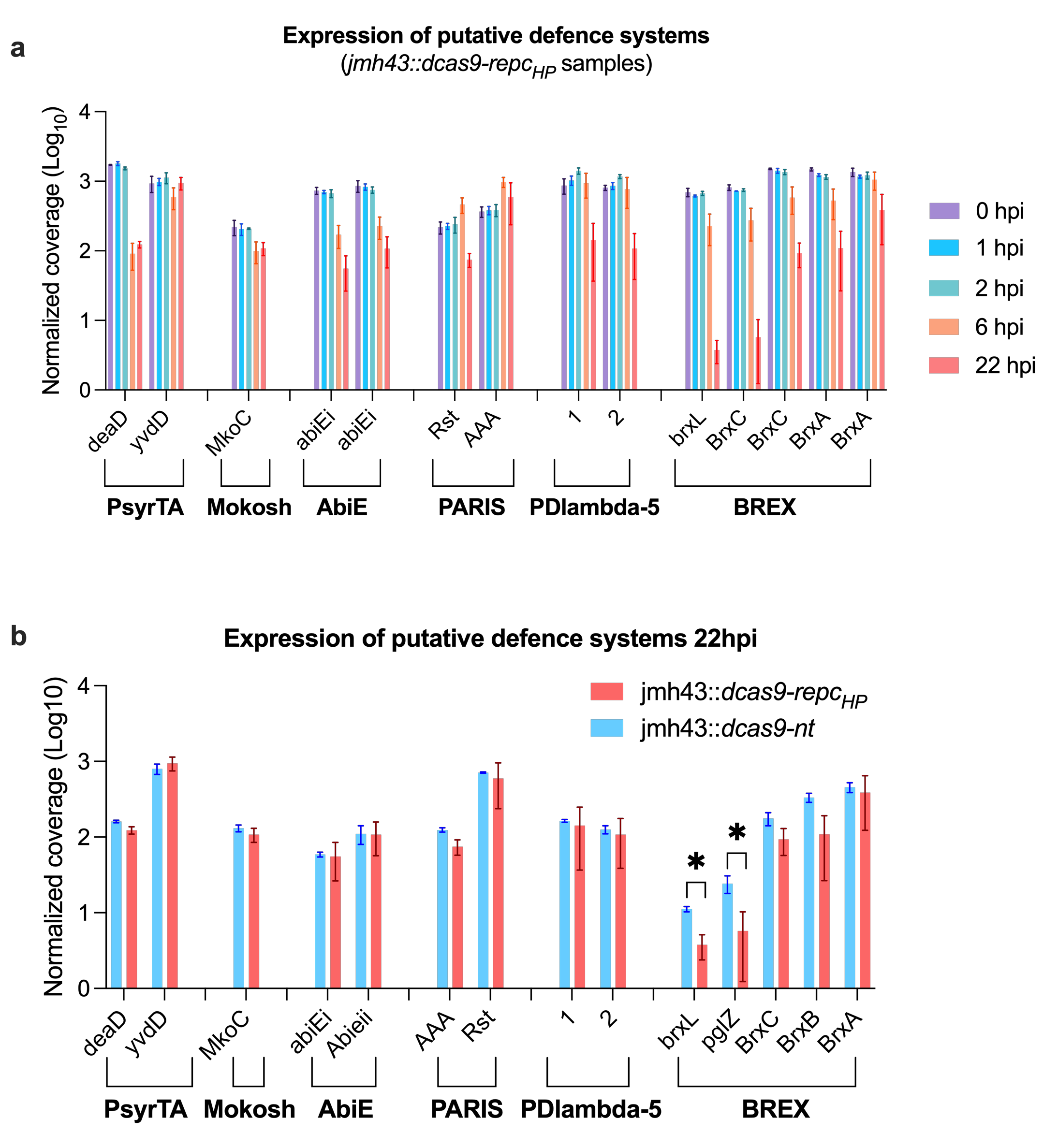

Supplement: S14 Fig — (a) Normalized transcriptomic coverage of the putative anti-phage defense systems of jmh43::dcas9-repcHP at different timepoints after IPTG-induced repcHP silencing. Coverage is normalized by total reads per sample and gene length. (b) Normalized transcriptomic coverage of the putative anti-phage defense systems of jmh43::dcas9-repcHP and jmh43::dcas9-nt at 22 hpi indicating the significant differences in expression calculated by the differential expression analysis. This was the only time point where significant differences in expression were detected for these genomic regions. The individual quantitative values underlying this figure can be found in the S15 Data file also available at: https://figshare.com/s/3ff18cc2f6cc1edab0ae. (TIFF) [file pbio.3002787.s014.tiff]

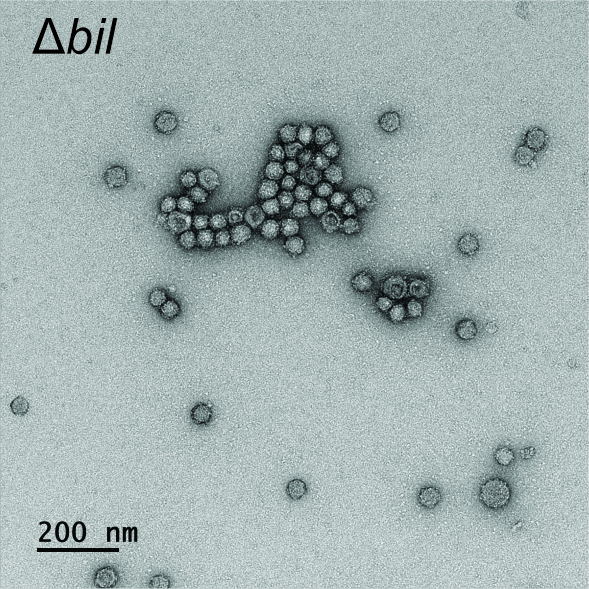

Supplement: S15 Fig — Negative staining and TEM microscopy of a mutant of the jmh43 strain where the putative bil defense system was deleted (jmh43Δbil). (TIF) [file pbio.3002787.s015.tif]
