# Supplementary material for: Incomplete lytic cycle of a widespread Bacteroides bacteriophage leads to the formation of defective viral particles
Source: PLoS Biol. 2025 Mar 31;23(3):e3002787. doi: 10.1371/journal.pbio.3002787 (PMC12135933; doi:10.1371/journal.pbio.3002787)
Supplement: Attachment — Submitted filename: Response to reviewers.PBIOLOGY-D-24-02179R3.pdf [file pbio.3002787.s041.pdf]

Response to reviewers

PBIOLOGY-D-24-02179R1 - "Incomplete lytic cycle of a widespread *Bacteroides* bacteriophage leads to the formation of defective viral particles"
Vendrell-Fernández *et al*.

Reviewer #1:

This manuscript describes investigation into certain aspects of lifestyle of Hankyphage - a temperate virus infecting Bacteroides, one of the most abundant and prevalent bacteria in the human gut. Since current knowledge on Bacteroides phages is limited, and the phage in question employing a somewhat unusual replicative transposition mechanism for its replication, the results presented in this manuscript present considerable interest to the human microbiome research community, as well as to phage biologists.

We thank the reviewer for their interest in our work.

Manuscript structure:

Results presented under the sub-section "Abnormal hankyphage virion assembly is partly recovered by repCHP silencing" invalidate preliminary conclusions that the reader might arrive to while reading the previous sub-section "Production of hankyphage virions does not lead to detectable lysis nor transduction". Therefore I suggest reversing the order in which results are presented and merging both subsections into one.

We agree, and we have now inverted the presentation and fused the two sections, indeed improving the flow of the article. More specifically:

• We moved the results of the section *"Production of hankyphage virions does not lead to detectable lysis nor transduction"* to the end of the section *"Abnormal hankyphage virion assembly in jmh43 is partly recovered by repc$_{HP}$ silencing"*:

Line 358: "Given the presence of some tailed virions in jmh43::*dcas9-repc$_{HP}$* lysates, we decided to test their infectivity. However, plaque assays using jmh43::*dcas9-repc$_{HP}$* lysates yielded no plaques on our HP-free strains nor jmh43ΔHP…"

• Moreover, we moved some results on the absence of lysis to the second results section *"Hankyphage virions are spontaneously produced by B. thetaiotaomicron isolates"* in order to streamline the fused section:

Line 192:" Previous studies reported a lack of plaque formation by hankyphage p00 on *P. dorei* naïve *strains* (Benler et al., 2018). Consistently, none of the lysates of our collection of HP+ *B. thetaiotaomicron* strains yielded plaques on any of our HP-free strains nor on the jmh43 strain cured from its hankyphage (jmh43ΔHP). To test if we could increase virion production and maximize the possibility of new infection events, we focused on the genetically amenable *B. thetaiotaomicron* jmh43 strain, which carries a hankyphage highly similar to the hankyphage p00 (IGS = 99%) (**Fig 1a**)."

• We also added a final paragraph to summarize the results of the section *"Abnormal hankyphage virion assembly in jmh43 is partly recovered by repc$_{HP}$ silencing"*:

Line 387: "Overall, our results show that the *B. thetaiotaomicron* jmh43 hankyphage spontaneously produces defective virions. However, despite the presence of complete virions after *repc*$_{HP}$-silencing, jmh43 hankyphages are non-infective in our laboratory conditions »

• We also presented the results corresponding to hankyphage packaging in a new section:

Line 575: '**Defects in hankyphage genome packaging *in vitro***'.

Specific comments:

- line 126: "Seven B. thetaiotaomicron strains out of the 22 (32%) hankyphage-like prophages, hereafter called HP+ strains..." - this is unclear, please revise

Indeed, one word was missing. This has been amended as follows:

Line 126: "Seven *B. thetaiotaomicron* strains out of the 22 (32%) **contained** hankyphage-like prophages, hereafter called HP+ strains..."

- line 387: "The law abundance..." - low abundance?

Thank you for spotting this typo, which is now corrected.

Line 341: "The **low** abundance…".

- line 415: to me this is more indicative of virion damage during PEG precipitation, partial ejection of DNA, and its degradation during the subsequent DNase treatment. Please consider this as an alternative explanation. Also trying alternative methods to concentrate/purify virions could be a great addition to this work.

The reviewer raised an important point. To address it, we used a PEG-free ultracentrifugation method to concentrate the capsids, and we can still observe an enrichment of the left-hand side of the hankyphage genome in all hankyphage strains (See new **Figure 6c**). The difference between the left-end:right-end copies of the hankyphage genome is still greater in PEG-precipitated capsids than in ultracentrifuged capsids. This suggests that, indeed, at least part of the uneven hankyphage genome coverage could be due to virion damage during PEG precipitation, besides a packaging defect.

We therefore amended our conclusions to include these observations as follows:

• We added a new **Figure 6c** depicting the ultracentrifugation data and placed the PEG-precipitation data as a supplementary **S9 Figure:**

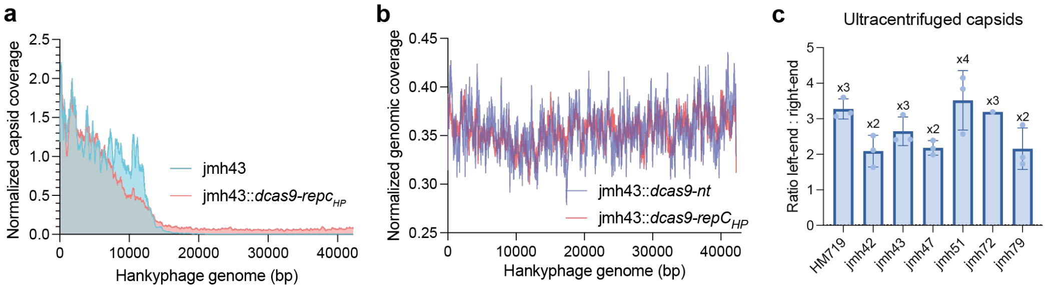

Line 422: **c.** Bar plot showing the ratio of hankyphage genome right-end and left-end copies quantified by ddPCR on ultracentrifuged capsids of HP+ strains.

• We introduced an explanation for the differences in the PEG-precipitated and the ultracentrifuged data in the results section:

Line 411: "To check that these differences were not due to virion damage during PEG concentration, we performed the same analysis after phage ultracentrifugation and found the same trend, albeit to a lesser extent (**Fig 5g**). Altogether these results suggest that the hankyphages in all HP+ *B. thetaiotaomicron* strains of our collection have a packaging defect or are unstable in laboratory conditions. »

• We introduced an alternative explanation for the skewed ratio of hankyphage right-end:left end copies in the results section:

Line 405: "Overall, our data shows that the jmh43 hankyphage DNA packaging is initiated at the left end of the phage but is randomly interrupted in the first 15 Kb or ejected from the capsid, with only a small fraction of capsids being fully packaged."

• In the discussion section: we introduced the alternative hypothesis, in addition to packaging defect, of a possible capsid fragility that may result in DNA ejection:

Line 500: "These observations suggest an incomplete capsid maturation process resulting in either partial DNA packaging or DNA ejection and its subsequent degradation upon DNAse treatment. Incomplete capsid maturation might also prevent tail-capsid attachment, which could explain the high frequency of detached capsids and tails in the *repc*$_{HP}$ silenced strain. "

• We added a methods section for the ultracentrifugation of the capsids:

Line 1314: "**Capsid concentration by ultracentrifugation**"

- line 986: ref 10 looks corrupted

Indeed, we apologize, and the reference has been corrected as follows:

Line 1000: "10. Benler S, Cobián-Güemes AG, McNair K, Hung SH, Levi K, Edwards R, et al. A diversity-generating retroelement encoded by a globally ubiquitous Bacteroides phage. Microbiome. 2018;6: 1–10. doi:10.1186/s40168-018-0573-6"

Please see also the section included at the end of our responses to reviewer #2 "*Other corrections introduced in the revised version of the manuscript*"

Reviewer #2:

Vendrell-Fernández et. al describe the characterization of a lysogenic Hankyphage in Bacteroides thetaiotaomicron. Using of variety of genetic, genomic, and biochemical techniques, they demonstrates the lysogen is spontaneously induced in regular laboratory culture. However, the resultant phage is seemingly defective, potentially due to incorrect virion assembly or genomic packaging. Diversity generating retroelements as well as a master repressor were identified in the phage and bioinformatic analysis revealed its prevalence in Bacteroides strains despite its inactivity in vitro. Altogether, the manuscript is a compelling set of negative data that points to the mysterious nature of Bacteroides phages that often do not follow the same set of rules as their better studied Proteobacteria and Firmicutes counterparts.

Major Comments

-Line 363 to 407 - The evidence that other prophages within the B. theta strain are not affecting Hankyphage production and maturation could be strengthen. The lack of DNA packaging in incomplete virions as well as the lack of proteins via mass spectroscopy does not show that the other prophage are not contributing to the incomplete pieces seen via TEM. Showing that transcription of these other prophage genes genes is not occurring would be stronger evidence that these components are only from the hankyphage. By the same token, it would be interesting to assess in the HP knockout strain if host genes are differentially expressed or if another prophage engages in transcription in the absence of HP. Even in the reported RNAseq dataset in Figure 3B, it would be important to note if knockdown of the repC repressor alters transcription of any genes outside of HP. For example, phage structural protein production could trigger a phage defense system that interferes with virion assembly.

We thank the reviewer for their comments and suggestions. Our RNAseq data shows that, in contrast to the hankyphage, we can detect the expression of only some genes of these prophages and not of their full genome (see new **S7 Fig** below). These results suggest that these other prophages are unlikely to produce virions in our experimental conditions, a conclusion that is corroborated by the lack of detection of their viral proteins by mass-spectrometry.

- We have added a new **S7 Fig** showing the expression of the different putative prophage-like regions:

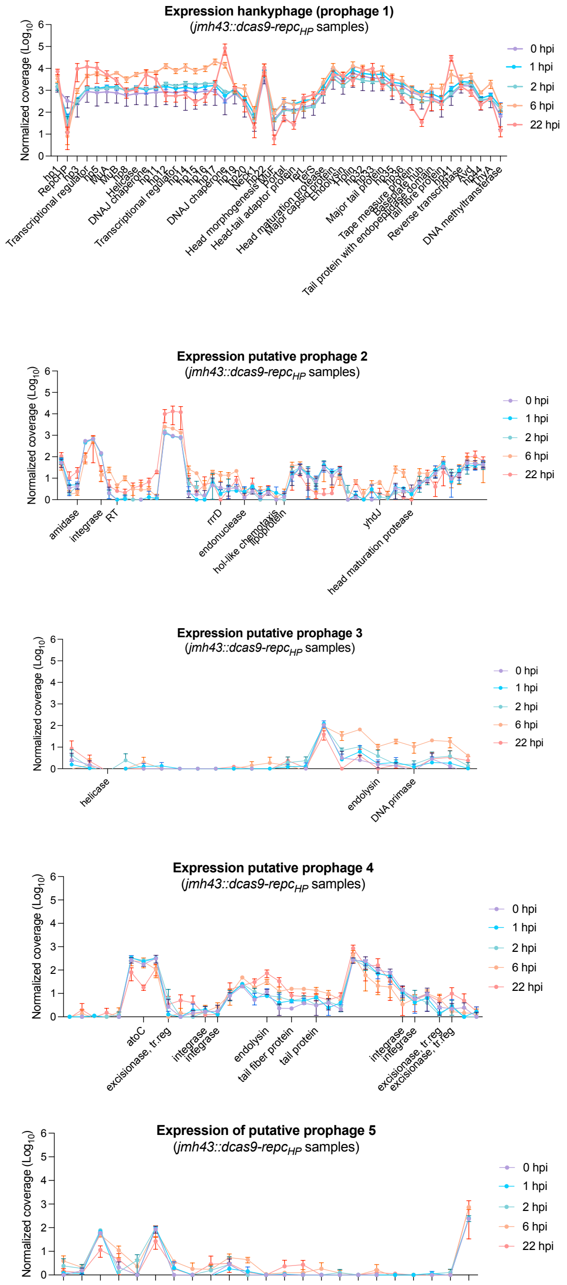

Line 1304: **S7 Fig. Expression of *B. thetaiotaomicron* jmh43 putative prophages.** Normalized transcriptomic coverage of the hankyphage genome and other putative prophages of jmh43::*dcas9-repc*$_{HP}$ at different timepoints after IPTG-induced *repc*$_{HP}$ silencing. Coverage is normalized by total reads per sample and gene length values of 0 were converted to 1 for their visualization with a Log_{10} scale.

- We now explicitly mention the expression of other putative prophage regions within the results section:

Line 349: "Our RNAseq data detected expression for only some genes of these prophages and not of the full genome – in contrast to the hankyphage were all genes were expressed (**S7 Fig**)."

- Please note that we have corrected two typos in the text, as there are four more other putative prophages in the jmh43 genome (instead of five) and six putative defense systems (instead of five):

Line 48: "The jmh43 strain encodes four other predicted prophages, which can be potentially induced and confound results (**S5 Table**).

Line 517: "Six anti-phage defense systems (**S8 Table**) could also be detected in the genome of jmh43..."

Similarly, all putative defense systems genes identified using DefenseFinder [47] are expressed at all timepoints with no significant differences in terms of gene expression between the *repc$_{HP}$*-silenced and the nt-control conditions (see new **S14 Fig**). Exceptionally, at timepoint 22hpi, two genes of the putative BREX systems were significantly downregulated (plgZ and BrxC) in the *repc$_{HP}$*-silenced strain compared to the control strain (see new **S14b Fig**), suggesting that hankyphage expression might downregulate this putative defense system.

We nonetheless agree with the reviewer that a defense system might still be responsible for the improper assembly of the Hankyphage. Defense systems can be activated at the protein level without being upregulated. In addition, we cannot exclude that unknown defense systems might be carried by other prophages, or other genetic elements, among the genes for which some expression is detected. Investigating this hypothesis would however require a lot of additional work and we believe this falls outside the scope of our article.

• To provide more information on the expression of the defense systems we have included a new supplementary **S14 Fig**:

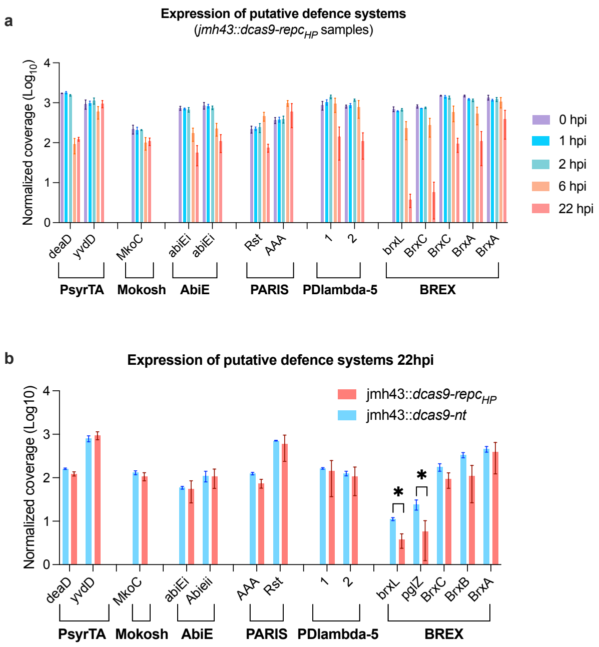

Line 1347: **S14 Fig. Expression of putative anti-phage defense systems in *B. thetaiotaomicron* jmh43. a.** Normalized transcriptomic coverage of the putative anti-phage defense systems of jmh43::*dcas9-repc$_{HP}$* at different timepoints after IPTG-induced *repc$_{HP}$* silencing. Coverage is normalized by total reads per sample and gene length. **b.** Normalized transcriptomic coverage of the putative anti-phage defense systems of jmh43::*dcas9-repc$_{HP}$* and jmh43::*dcas9-nt* at 22 hpi indicating the significant differences in

expression calculated by the differential expression analysis. This was the only timepoint where significant differences in expression were detected for these genomic regions.

• We also provide this information in the discussion section and its possible implications:

Line 517: “Six anti-phage defense systems (**Table S8**) could also be detected in the genome of jmh43 [1] and were transcriptomically active according to our RNAseq data (**S13 Fig**). »

Line 524: We cannot exclude that some of the other defense systems detected, or that unknown anti-phage systems might be responsible for the defects in hankyphage assembly. A recent study demonstrated how defense systems carried by prophages can block the lytic cycle and release of competing prophages present in the bacteria [2]. A future direction will be to delete defense systems and prophages in a *B. theta* host to test this hypothesis.

Beyond the putative prophages and defense systems, the expression of several hundred genes were detected to be significantly altered. However, given the high number of hypothetical genes, we could not perform an informative KEGG analysis to bring forward the possible cellular effects of *repC*$_{HP}$-silencing.

• We have included this information in the discussion section and backed it up with a supplementary table (**S10 Table**) containing the significantly upregulated or downregulated genes at the different timepoints.

Line 530: « Moreover, beyond the putative prophages and defense systems, the expression of several hundred genes were detected to be significantly altered in the jmh43::*dcas9-repc*$_{HP}$ strain compared to the control strain at 6 hpi and 22 hpi (**Table S10**). Even though the high number of hypothetical genes did not allow for an informative KEGG analysis, these results highlight the possibility of other cellular mechanisms interfering with hankyphage assembly or being an effect of hankyphage induction. »

Line 1395: “**Table S10. All differentially expressed genes in jmh43::*dcas9-repc*$_{HP}$ compared to jmh43::*dcas9-nt* strain.** Table indicating the Log2fold change and p values of the differential gene expression analysis comparing the jmh43::*dcas9-repc*$_{HP}$ compared to jmh43::*dcas9-nt* at all the different timepoints (0, 1, 2 6 or 22 hpi). Differentially expressed genes were determined using a cutoff of a minimum of 1 Log2 fold change and a p-adjusted value < 0.05.”

Finally, whereas investigating the expression of other prophage regions in the absence of the hankyphage could be interesting, we would like to argue that, considering that we focused the induction of the hankyphage itself, this would be beyond the scope of our manuscript.

-Figure 4b - Only having one replicate is insufficient to make a fair comparison between the nt and repC dCas9 strains. One would expect transposition to be a stochastic event and additional replicates would be required to show that the extent of transcription is truly greater in the repC knockdown and not due to a jackpot effect in the nt control. I would suggest repeating this experiment with several more replicates to show this is a consistent occurrence.

We agree and we have now sequenced two more biological replicates of each strain at every time point, and we still observe an increase in the frequency of transpositions at different locations in the *repc*$_{HP}$-silenced strain compared to the control strain.

• We have modified **Figure 4b** and its caption accordingly:

Line 278: **"b.** Quantification of transposition events of in jmh43::*dcas9-repc*$_{HP}$ and jmh43::*dcas9-nt* at different timepoints (three biological replicates)."

• Additionally, we have also modified the method for quantification of transposition events without using paired-end information, which varies across sequencing runs based on the insert size. We have now mapped the new junctions more precisely, without the need of a window-based approach.

Line 763: **Quantification and mapping of transposition events**
Raw reads from genomic DNA were mapped to the jmh43 HP prophage using bwa mem v. 0.7.17-r1188 [3], following delimitation of the prophage ends using capsid data. If the prophage had integrated into a different chromosomal region, reads at the ends would be clipped. Soft-clipped reads were extracted with the Pysam package (https://pysam.readthedocs.io/en/latest/index.html). The clipped regions (≥ 15 nt) were subsequently searched into the jmh43 bacterial chromosome using blastn (-task blastn-short -qcov_hsp_perc 100 -perc_identity 100). Clipped reads that were mapped into multiple genome locations were discarded (<1%). To quantify new junction locations, we considered only reads clipped at the start of the HP and recorded the last chromosomal position of each aligned read. The plot of integration spots across the jmh43 genome was generated with pyCirclize [4].

• This has resulted in a higher number of transposition events than in the previous version of the manuscript but that is consistent across the three replicates. We have hence corrected the values in the text of the results section:

Line 269: "These insertions culminated at an average of 2504 transposition events at 22 hpi compared to an average of 40 events in the control (**Fig 4b**)."

-Throughout - It is claimed that hankyphages as a whole are incapable of infecting and transducing in laboratory settings, however you only show data for a single phage for the vast majority of the paper (jmh43). These claims can only be applied to jmh43 and consistently applying them to hankyphages across B. theta or Bacteroides lacks substantial evidence. I suggest softening generalized claims about Hankyphage to focus on jmh43 or performing the experiments to include 2+ more phage from the collection of 7 that was obtained.

We agree with the reviewer and have toned down the claims on hankyphage infectivity:

• We have changed the title of one of the results sections to indicate that these are observations performed mostly in jmh43:

Line 303: "Abnormal hankyphage virion assembly **in jmh43** is partly recovered by recovered by *repc*$_{HP}$ silencing"

• Moreover, as suggested by the first reviewer, we have placed the results of the section *"Production of hankyphage virions does not lead to detectable lysis nor transduction"* under *"Abnormal hankyphage virion assembly in jmh43 is partly recovered by recovered by repc*$_{HP}$ *silencing"*, indicating that the results on the absence of infectivity are solely on the jmh43 hankyphage.

• Additionally, we have included evidence showing that the non-induced hankyphages from our collection do not infect naïve *B. thetaiotaomicron* strains.

Line 192: "Previous studies reported a lack of plaque formation by hankyphage p00 on *P. dorei* naïve strains (Benler et al., 2018). Consistently, none of the lysates of our collection of HP+ *B. thetaiotaomicron* strains yielded plaques on any of our HP-free strains nor on the jmh43 strain cured from its hankyphage (jmh43ΔHP). »

We did not perform additional $repc_{HP}$-silencing nor an insertion of the erythromycin resistance gene to test transduction in the other hankyphages (some of them such as the jmh51 hankyphage were genetically unamenable with available approaches). Nevertheless, we consider that the absence of plaque formation observed with overnight non-induced lysates are consistent with the claim that the hankyphages from our collection are not infective in laboratory conditions.

-While likely beyond the scope of the manuscript, it would be interesting to see if: 1) Transduction could be observed in the mouse gut; 2) Transduction could be observed in ex vivo conditions, such as cecal extracts; 3) If different media types would yield viable virions, especially those that better resemble the ionic strength and pH of the gut environment where (presumably) these phages are active.

We agree with the reviewer in the interest of these experiments. As pointed out by the reviewer, we consider animal experiments to be outside the scope of the article, we have however performed transduction tests in fecal extracts using the lysates of jmh43-*HP-Erm* and jmh43::*dcas9-repc-HP-Erm,* in which an erythromycin resistance gene was inserted in the hankyphage genome. These experiments did not yield any transduction events.

• We have included the results of these transductions in the results section:

Line 384: "Additionally, to better imitate the gut physiological conditions which might facilitate phage infection, we performed the same transduction experiment using mouse fecal extracts, but this did not yield any transductants neither."

• Moreover, we have also included the protocol for the transduction in the methods section.

Line 634: "Overnight cultures were diluted to an OD600=0.05 and grown anaerobically in 5mL cell culture tubes at 37°C for 2h until exponential phase. 1mL of cells were then pelleted and were either resuspended with 200µl of the phage lysate or in a 1:1 ratio mixture of the phage lysate and mouse faecal filtrate. These were then incubated anaerobically and statically at 37°C for 2 hours. The mixture was then plated on erythromycin-supplemented BHIS plates and grown for 48°C at 37°C anaerobically. The colonies obtained were re-streaked on erythromycin-supplemented BHIS and single colonies were tested by PCR for the presence of the hankyphage and the *ermG* gene."

Concerning point **3)** among the induction conditions that we tried, there is a representation of ionic strengths and pHs found in the gut environment. For instance, the Gut microbiota medium (GMM) and the MiPro medium both have a pH 6-7, and a similar ionic strength to the large intestine (Pye et al., (1990). Gastrointestinal intraluminal pH in normal subjects and those with colorectal adenoma or carcinoma. Gut, 31(12), 1355–1357). Though we did not look at these virions under the electron microscope, these lysates did not yield plaques on *B. thetaiotaomicron* naïve strains, suggesting that they were defective even when grown in these conditions

Though this was stated in the previous version of the manuscript (within the results section "Hankyphage virions are spontaneously produced by *B. thetaiotaomicron* isolates"), we have rephrased this sentence for clarity as follows:

Line 204: None of these conditions revealed a significant increase in hankyphage virion production (**Fig 2b**) and the virions obtained did not lead to any plaque formation on naïve *B. thetaiotaomicron* strains (jmh43 ΔHP and jmh44).»

Minor Comments

-Line 242 - Different citation format than the rest of the manuscript.

This has been corrected

Line 241: "…target any *B. thetaiotaomicron* genes [23] at the …"

-Line 449 - grammatical issue. Should be "(Sig 11 Fig), suggesting)"

This has been corrected by splitting the sentence into two separate sentences as follows:

Line 448: "Moreover, analysis of the coverage pattern of hankyphages in metaviromes [6] did not reveal a drop in coverage toward the 3' end of the hankyphage genome **S12 Fig**). This suggested that the defect in capsid DNA packaging that we observe in our laboratory conditions does not occur in vivo."

-Figure 1b - State total number of cultures used to determine nucleotide diversity

This is now stated in the caption and in the text as follows:

- In figure 1b caption:

Line 144: "**b.** Nucleotide diversity (π) of the hankyphage VR region for individual overnight cultures of HP+ *B. thetaiotaomicron* strains"

- In the text:

Line 155: "we analyzed sequencing data obtained from total DNA extracted from single overnight cultures."

-Figure 4c - Why is there a dip in virions at 2 hours for one of the jmh43::dcas9-repCHP replicates?

Thank you for pointing this out. We repeated the ddPCR of all the samples at 2hpi and no longer observe a drop in virions a timepoint 2 hpi. This drop could have been due to a pipetting error. The figure has been amended accordingly.

Other corrections introduced in the revised version of the manuscript

- **Improved hankyphage annotation:**

  -We manually curated the hankyphage annotation using FoldSeek for the low confidence hits. This extra step of curation was added to the materials and methods section:

  Line 701: "Genes with low confidence were manually curated using Foldseek [63]."

  - This method has resulted in several changes, notably, the previous minor tail protein is now the tail fiber protein, and the DNA polymerase is a putative DNAJ-like protein. This has been amended accordingly in **Figure 1a**, **Figure 3b** and **S3b Fig.**

  -Additionally, we annotated the hypothetical genes, which allows their identification easily in the mass spectrometry and RNAseq data. The annotation of the hypothetical genes has been incorporated to the caption of Figure 1a:

  Line 139: « Gene annotations for genes that had a predicted function are indicated in black whilst hypothetical genes are numbered and annotated in gray. »

  -Moreover, we amended the text accordingly:

  Line 132: Their encoded hankyphages were generally conserved and mainly differed in the MuA-like transposase, putative tail fiber and DGR reverse transcriptase (**Fig 1a**).

  Line 160: This showed that the DGR diversifies the hankyphage tail fiber gene in our growth conditions.

  -The adjustments in the hankyphge genome annotation resulted in the correction of some gene boundaries. Hence, we re-ran the RNAseq differential expression analysis with this adjusted annotation. This resulted in very similar results with slightly different values, that we amended in **Figure 3b**, **S3b** and **S9 Table** accordingly. Still, some genes that were previously non-differentially expressed, were now differentially expressed, including the MuF-like gene. Hence, we removed the paragraph within the discussion section about the possible *repc*$_{HP}$ -independent regulation of this gene from the discussion.

- **RepC capitalization*:*** We corrected the name of the Mu phage repressor c (Repc) that was previously written as 'MuRepC'. We also removed the capitalized 'C' for a lower 'c' in the hankyphage *repc*$_{HP}$ gene to follow the convention of writing of genes.

- **Figure 4d:** We corrected a formula error in the CFU counts, which had resulted in abnormally high values. The results now depict values typical of *Bacteroides* cultures.

- **Figure 5a:** Added zoomed sections of the TEM micrographs with measurements for their better interpretation.

- **S6 Fig:** Corrected the measurements of the capsids of tailed hankyphages due to a scale error when using FIJI. The new values are lower than in the previous version of the manuscript but still significantly different to the jmh43 particles.

- **Clarified supplementary Tables S6, S8 and S9**: We have included more information precising the table titles and the colouring.

- **Scientific nomenclature precisions**

-Change of 'lytic' for 'virulent' phages:

-Lines 78: leading to the identification of new viral groups of both virulent and temperate bacteriophages [1]

-Line 84: *Bacteroides* are specifically targeted by various virulent bacteriophages [5–7]

-Changed to be more precise the transposition effects of transposable phages:

Line 98: Additionally, hankyphages contain a putative transposase and may replicate via replicative transposition [6], contributing to the *Bacteroides* genetic diversity through various genomic rearrangements'

-We substituted 'siphovirus-like' for 'tailed phages'.

Line 308: 'TEM data presented in the initial description of the hankyphage p00 were suggestive of a tailed phage...'
